# One clot after another in COVID-19 patient: diagnostic utility of handheld echocardiogram

**DOI:** 10.1093/omcr/omaa141

**Published:** 2021-02-15

**Authors:** Gini Priyadharshini Jeyashanmugaraja, Evgeny Shloknik, Deborah Tosin Akanya, Kristin Stawiarski, Christopher Winterbottom, Stuart Zarich

**Affiliations:** 1 Department of Internal medicine, Yale New Haven Health Bridgeport Hospital, Bridgeport, CT, USA; 2 Department of Cardiology, Yale New Haven Health Bridgeport Hospital, Bridgeport, CT, USA; 3 Department of Pulmonology and Critical Care, Yale New Haven Health Bridgeport Hospital, Bridgeport, CT, USA

## Abstract

A 63-year-old woman was admitted with severe respiratory distress requiring mechanical ventilation and shock requiring vasopressor support. She was found to have COVID-19 pneumonia. Focused cardiac ultrasound performed for evaluation of shock was significant for right ventricular dilation and dysfunction with signs of right ventricular pressure overload. Given worsening shock and hypoxemia systemic thrombolysis was administered for presumed massive pulmonary embolism with remarkable improvement of hemodynamics and respiratory failure. In next 24 h patient’s neurologic status deteriorated to the point of unresponsiveness. Emergent computed tomography showed multiple ischemic infarcts concerning for embolic etiology. Focused cardiac ultrasound with agitated saline showed large right to left shunt due to a patent foramen ovale. This was confirmed by transesophageal echocardiogram, 5 months later. This case highlights strengths of focused cardiac ultrasound in critical care setting and in patients with COVID-19 when access to other imaging modalities can be limited.

## INTRODUCTION

We present a patient with COVID-19 infection presenting with respiratory failure and shock. Our case report highlights the strengths of focused cardiac ultrasound in differential diagnosis of shock and paradoxical embolism, especially valuable in patients with COVID-19 when access to other imaging modalities can be limited.

## CASE REPORT

**Figure 1:**
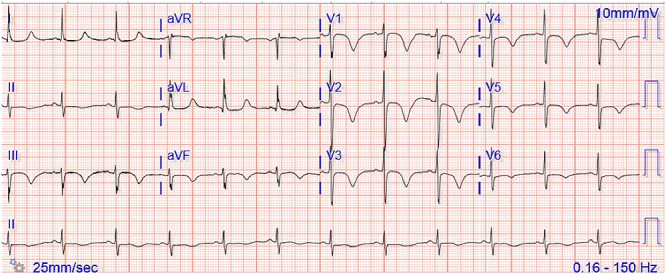
Initial electrocardiogram showing sinus rhythm with rate of 70 bpm, tall R wave in V2 and symmetric T wave inversions in precordial leads.

A 63-year-old woman with the history of hypertension presented to the emergency department during COVID-19 pandemic with significant shortness of breath on minimal exertion and fatigue for 3 weeks. Patient was hypoxic to 74% and tachypneic to 30 breaths/min and required endotracheal intubation. Physical exam was unremarkable. Shortly, thereafter, she became persistently hypotensive requiring vasopressor support. Electrocardiogram revealed sinus rhythm with rate of 70 bpm, tall R wave in V2 and symmetric T wave inversions in precordial leads (Fig. 1). Chest radiograph post intubation showed bilateral patchy infiltrates. Initial severe acute respiratory syndrome-coronavirus-2 (SARS-CoV-2) PCR test was negative while the second PCR for SARS-CoV-2 done 24 h later returned positive.

Initial troponin T was 0.07 ng/ml with maximum at 0.22 ng/ml (reference range < 0.01 ng/ml) and NT-proBNP was 12 945 pg/ml. Elevated white blood cell count (21.1 × 10^3^/μl), creatinine (1.45 mg/dl), C-reactive protein (25.9 mg/dl) and ferritin (546 ng/ml) and D- Dimer 4.34 (ref range ≤ 0.63 mg/l) was noted. Serial ECGs remained stable.

Patient remained persistently hypoxic with PaO_2_/FiO2 ratio of 85 which along with CXR findings is suggestive of severe acute respiratory distress syndrome (ARDS). Central venous oxygen saturation of 62% suggested the possibility of mixed shock.

**Figure 2:**
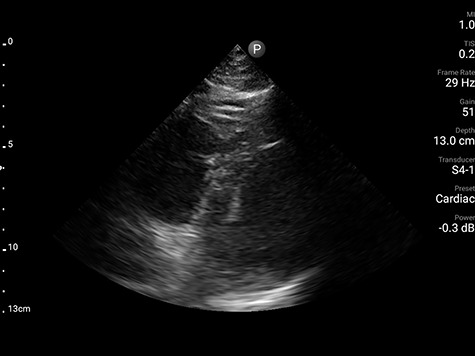
Parasternal short-axis view demonstrating dilated right ventricle (star) with flattened septum (arrow).

Computed tomography angiogram of chest was deferred due to hemodynamic instability and refractory hypoxemia. Systemic anticoagulation with heparin and broad-spectrum antibiotics was initiated. Due to institutional COVID-19 policy for transthoracic echocardiography to minimize personnel exposure, focused cardiac ultrasound (FoCUS) was performed, which revealed mildly increased right ventricular cavity size with flattened septum in systole and diastole along with bowing of interatrial septum toward the left supporting ECG findings of right ventricular strain (Fig. 2, Supplementary video 1). Due to suspected massive pulmonary embolism (PE) alteplase 50 mg was given intravenously over 120 min. This resulted in significant improvement of hemodynamics, perfusion indices and oxygenation within next 12 h to the extent that hypotension resolved despite being on high PEEP protocol for ARDS.

**Figure 3:**
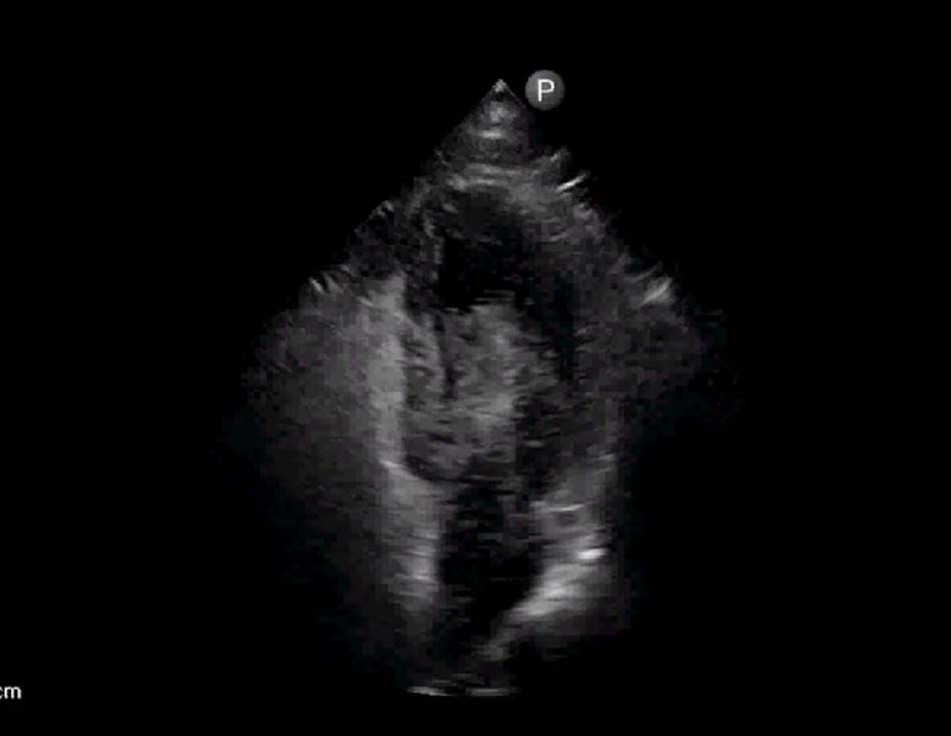
Apical four chamber view with early right to left shunt by agitated saline study.

After 24 h, patient was noted to be unresponsive despite being off sedation. Urgent computed tomography (CT) of head revealed multifocal infarcts of both cerebral and cerebellar hemispheres with associated mass effects concerning for acute ischemic infarcts. Follow-up CT in 12 h revealed hemorrhagic transformation and anticoagulation with heparin was held. Due to concern for cardioembolic phenomenon, FoCUS with agitated saline was done that showed early right to left shunt consistent with patent foramen ovale (PFO) (Fig. 3, Supplementary video 2). Doppler ultrasound of legs did not reveal deep venous thrombosis (DVT). No evidence of atrial fibrillation was noted on prolonged cardiac monitoring. Antiphospholipid antibody syndrome workup was negative.

**Figure 4:**
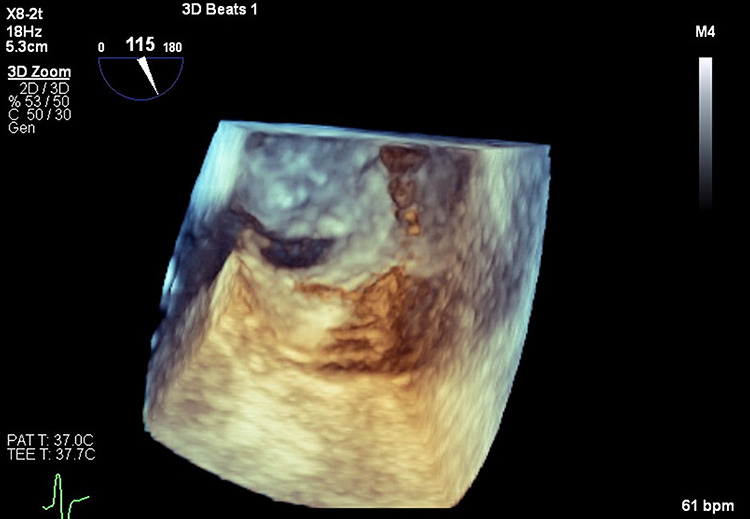
Three-dimensional transesophageal echocardiography with PFO in interatrial septum.

**Figure 5:**
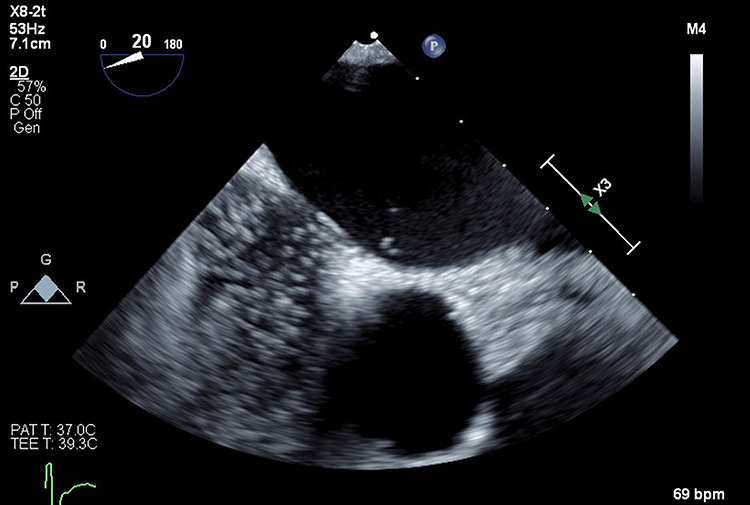
Transesophageal echocardiography. Agitated saline study with bubbles crossing from right atrium to left atrium through PFO.

Patient remained intubated for 20 days due to poor mental status but was eventually successfully liberated from mechanical ventilation. She was discharged on apixaban 5 mg BID for PE. After 5 months of continuous physical, neurological and nutritional improvement, TEE was done which confirmed the PFO (Figs. 4 and 5). She was referred for PFO closure.

## DISCUSSION

Shock in COVID-19 patients requires careful assessment. Distributive shock from inflammatory response to SARS-CoV-2 is common, but other types of shock including cardiogenic, obstructive (including tamponade and tension pneumothorax), as well as mixed shock should be considered. Myopericarditis, stress cardiomyopathy, and myocardial infraction (MI) should be in the differential for cardiogenic shock in such patients. A high degree of clinical suspicion is required to diagnose obstructive shock from PE given significant hypoxemia in severe COVID-19 cases due to ARDS [1]. Classic ECG changes of right ventricular strain S1Q3T3 are insensitive. On the other hand, simultaneous T wave inversions in inferior and precordial leads with maximum amplitude of T wave in V1–V2 was previously reported, as sign of PE [2].

Hypercoagulability, both in macro and microvascular circulation, is important contributor to morbidity and mortality in COVID-19 infection, and our understanding of the disease is evolving rapidly. Macro-thrombotic events including acute MI, acute limb ischemia, stoke, DVT, PE as well as pulmonary intravascular coagulopathy with micro-thrombotic complications are described in [3]. Early diagnosis of these grave thrombotic complications in COVID-19 patients may improve outcomes substantially.

COVID-19 pandemic created a situation wherein healthcare workers safety and highest care to patients must be in delicate balance. Multiple institutions including American Society of Echocardiography implemented new policies to change use criteria and protocols for different imaging modalities to limit exposure of healthcare workers [4, 5]. FoCUS is valuable bedside tool and able to provide information on left and right ventricular systolic function, valvular abnormalities, pericardial effusion and volume status [6]. There are only limited reports on echocardiography and FoCUS use in COVID-19 patients [7]. Study by European Association of cardiovascular imaging showed 55% of patients with presumed or confirmed COVID-19 had abnormal echocardiography results which changed the management in one-third of the patients [8]. In a smaller study of 91 suspected or known COVID-19 patients, sonographers spent significantly less time using handheld ultrasound but still provided sufficient information for clinical team [9]. The major limitation of FOCUS is the diagnostic accuracy is dependent on the operator’s skills.

In our case, FoCUS was not only used for diagnosis of massive PE but also to diagnose the large interatrial shunt. Prompt diagnosis of massive PE and appropriate thrombolysis dramatically improved hemodynamics and oxygenation. Although stroke can be explained by prothrombotic state in COVID-19, embolic origin, particularly paradoxical embolism, needs to be ruled out [10]. FoCUS with agitated saline confirmed the diagnosis of interatrial right to left shunting as route for paradoxical embolism.

To conclude, FoCUS facilitated diagnosis of massive pulmonary thromboembolism and interatrial shunt that guided clinical management. High clinical suspicion for thrombotic complications in COVID-19 patients is required for prompt diagnosis. FoCUS is a helpful tool readily available at bedside with advantage of limiting exposure of healthcare workers.

## CONFLICT OF INTEREST STATEMENT

No conflicts of interest.

## FUNDING

Not applicable.

## ETHICAL APPROVAL

None required.

## CONSENT

We obtained written informed consent from the patient for the publication of this case report and the accompanying images.

## GUARANTOR

Stuart Zarich MD.

## Supplementary Material

Figure_2_Online_video_1_omaa141

Figure_3_online_video_2_omaa141
