# Corrigendum to : One clot after another in COVID-19 patient: diagnostic utility of handheld echocardiogram

**DOI:** 10.1093/omcr/omab070

**Published:** 2021-07-27

**Authors:** Gini Priyadharshini Jeyashanmugaraja, Evgeny Shkolnik, Deborah Tosin Akanya, Kristin Stawiarski, Christopher Winterbottom, Stuart Zarich

**Affiliations:** 1 Department of Internal medicine, Yale New Haven Health Bridgeport Hospital, Bridgeport, CT, USA; 2 Department of Cardiology, Yale New Haven Health Bridgeport Hospital, Bridgeport, CT, USA; 3 Department of Pulmonology and Critical Care, Yale New Haven Health Bridgeport Hospital, Bridgeport, CT, USA

When this paper first published, an author name was incorrectly given as ``Evgeny Shloknik''. This should have appeared as ``Evgeny Shkolnik''. This has now been corrected online

